# Room-temperature short-wavelength infrared Si photodetector

**DOI:** 10.1038/srep43688

**Published:** 2017-03-06

**Authors:** Yonder Berencén, Slawomir Prucnal, Fang Liu, Ilona Skorupa, René Hübner, Lars Rebohle, Shengqiang Zhou, Harald Schneider, Manfred Helm, Wolfgang Skorupa

**Affiliations:** 1 Institute of Ion Beam Physics and Materials Research, Helmholtz-Zentrum Dresden-Rossendorf, P.O. Box 510119, 01314 Dresden, Germany; 2 Technische Universität Dresden, 01062 Dresden, Germany

## Abstract

The optoelectronic applications of Si are restricted to the visible and near-infrared spectral range due to its 1.12 eV-indirect band gap. Sub-band gap light detection in Si, for instance, has been a long-standing scientific challenge for many decades since most photons with sub-band gap energies pass through Si unabsorbed. This fundamental shortcoming, however, can be overcome by introducing non-equilibrium deep-level dopant concentrations into Si, which results in the formation of an impurity band allowing for strong sub-band gap absorption. Here, we present steady-state room-temperature short-wavelength infrared p-n photodiodes from single-crystalline Si hyperdoped with Se concentrations as high as 9 × 10^20^ cm^−3^, which are introduced by a robust and reliable non-equilibrium processing consisting of ion implantation followed by millisecond-range flash lamp annealing. We provide a detailed description of the material properties, working principle and performance of the photodiodes as well as the main features in the studied wavelength region. This work fundamentally contributes to establish the short-wavelength infrared detection by hyperdoped Si in the forefront of the state-of-the-art of short-IR Si photonics.

One of the most pressing challenges in the realm of silicon photonics is the detection of light at short-wavelength infrared (SWIR) spectral range (viz. λ = 1,400–3,000 nm). This is of great interest for optical communications, spectroscopy, sensing, medical imaging, environmental monitoring and integrated optoelectronics^1^,^2^,^3^,^4^. Silicon is a high-quality semiconducting material, low-cost and the material par excellence of the complementary-metal-oxide-semiconductor (CMOS) technology, but it is optically transparent at photon energies below its 1.12 eV-band gap, which makes the sub-band gap optoelectronic response very challenging. Conventional Si photodetectors typically show peak-photoresponse between 700 and 900 nm with very low dark currents due to the high crystalline quality and excellent passivation properties of Si.

In the continuing quest of extending the Si photoresponse towards the SWIR spectral range, different strategies have been adopted. The integration of direct-band gap III–V compound semiconductors (GaAs, InAs) with Si gives rise to photodetectors with extended IR photoresponse^5^, but the lattice mismatch makes this approach difficult. Alternatively, Park *et al*. reported on the integration of a hybrid AlGaInAs-silicon evanescent photodetector operating at 1,550 nm using wafer bonding^6^, although this fabrication method still suffers from thermal expansion coefficient mismatch^7^. Photodetectors based on SiGe alloys^8^ and Si/SiGe quantum wells^9^ have also exhibited room-temperature SWIR photoresponse. However, a high temperature (≥700 °C) epitaxial growth of Ge is required, which might infringe upon the CMOS compatibility.

Another approach consists in extrinsic Si photodetectors. Sub-band gap photoresponse from Si photodetectors doped with group III or V impurities (Al, B, P, As) has been reported, but operation temperatures below 40 K are required, since such dopants introduce shallow impurity levels within the Si band gap that favor thermally ionized carriers^10^. Alternatively, the creation of dislocation centers and lattice point defects by ion bombardment has led to reduce the thermally generated carriers at the expense of low sub-band gap absorption coefficient (α ≈ 0.5 cm^−1^)^11^. Recently, published works involving avalanche Si photodetectors based on silicon-on-insulator (SOI) waveguides with a lateral p-i-n junction take advantage of combining the diode-avalanche regime and lattice defects introduced by low-dose inert ion implantation to enhance the defect-mediated SWIR photoresponse^2^. These results correspond to photodetectors with an impressive high speed detection of 20 Gbit s^−1^ at 1.96 μm. Unfortunately, this device proposal requires (i) absorption regions on the order of hundreds of micrometers in length due to the relatively weak interaction of sub-band gap light with lattice defects and (ii) high operating voltages between 15 V and 27 V for triggering the avalanche working regime, which compromises the operating voltages dictated by CMOS device design.

A different and promising alternative is the incorporation of chalcogens (S, Se, Te) at concentrations far above the solid solubility limit in Si (≈10^16^ cm^−3^)^12^,^13^,^14^. Dopant concentrations at least four orders of magnitude (≈10^20^ cm^−3^) above this limit have been introduced into Si by using non-equilibrium processing methods such as ion implantation followed by pulsed laser melting (PLM) or pulsed laser irradiation of Si, which has to be immersed in an atmosphere containing chalcogen atoms^12^,^13^. This results in the formation of an impurity band (IB)^15^ which induces a significant enhancement of the sub-band gap photoresponse. Additionally, this fact enables higher operating temperatures since chalcogens introduce deep double donor levels that significantly mitigate thermal carrier generation. This new class of hyperdoped materials exhibit the highest absorption coefficient (α ≈ 10^4^ cm^−1^) ever obtained for Si in the SWIR spectral range^12^,^13^. This value is also similar to the one reported for In_0.53_Ga_0.47_As and intrinsic Ge^16^,^17^ and in turn twelve orders of magnitude higher than that for intrinsic Si (α < 10^−8^ cm^−1^) at wavelengths longer than 1,550 nm^18^. Interestingly enough, chalcogen-supersaturated Si photodetectors fabricated by ion implantation followed by nanosecond PLM have only exhibited sub-band gap optoelectronic photoresponse at wavelengths as short as 1,250 nm^14^, and photoconductivity in the SWIR spectral range only at low temperature^13^. More recently, room-temperature 1,550 nm photoconductivity has also been reported on sulfur-supersaturated Si photodetectors electrically compensated with boron and fabricated by ion implantation followed by nanosecond PLM^19^, but a room-temperature SWIR chalcolgen-hyperdoped Si photodetector still remains elusive. In this context, alternative deep-level dopants, such as gold, have also been used for hyperdoping Si by ion implantation and PLM. Room-temperature sub-band gap photoresponse from Au-hyperdoped Si photodetectors with external quantum efficiencies of around 10^−4^ at 2,200 nm has been reported^3^. Similarly, room-temperature Zn-implanted Si waveguide photodiodes with responsivities of around 87 mA/W at 2.3 μm have recently been demonstrated^20^. As very fast diffusers in Si^21^, Au and Zn are still unappreciated elements in the mainstream CMOS-compatible device fabrication processes.

In this work, we report on the significant room-temperature SWIR photoresponse exhibited by Se hyperdoped Si p-n photodiodes fabricated by an alternative non-equilibrium processing method consisting of ion implantation and millisecond-flash lamp annealing (FLA). The success of the room-temperature SWIR Si photodiodes lies on the energy band engineering of Si at Se concentrations as high as 9 × 10^20^ cm^−3^ and the resulting high quality of the engineered material. In contrast to conventional liquid phase epitaxy induced by PLM, FLA in the millisecond range allows for solid phase epitaxy that has recently reported to provide chalcogen-hyperdoped Si materials of superior quality^22^. The resulting Si photodiodes render very low operating voltages and a strong room-temperature sub-band gap photoresponse at wavelengths as long as 1,600 nm. Additionally, Fourier transform infrared (FTIR) spectroscopy results predict that this room-temperature SWIR photoresponse might be extended up to 3,100 nm. These results clearly demonstrate the possibility of opening a broad palette of optoelectronic functionalities with versatile applications in the next generation of short-IR Si photonic systems.

## Results and Discussions

### Fabrication of Se-hyperdoped Si layer and its microstructural properties

Double-side polished <100> p-type Si substrates with resistivity of 1–10 Ω cm were implanted at room temperature with Se ions at fluences of 3 × 10^15^ cm^−2^, 6 × 10^15^ cm^−2^ and 9 × 10^15^ cm^−2^ with an implantation energy of 60 keV. This allows for a projected range of 50 nm and atomic Se concentrations of around 1.1%, 2.3% and 3.5% as experimentally verified by Rutherford backscattering spectrometry (RBS) measurements. Subsequently, implanted samples were flash-lamp annealed in N_2_ atmosphere with an energy of 33 J/cm^2^ for 1.3 ms. Prior to this annealing step, a preheating at 300 °C for 30 s was performed in order to reduce the internal strain during FLA^23^. Under these conditions – and in contrast to liquid phase epitaxy driven by laser annealing methods – solid phase epitaxy is induced allowing for single-crystalline hyperdoped Si free of extended defects. Moreover, Se segregation and diffusion is suppressed resulting in Se concentrations as high as 9 × 10^20^ cm^−3^ incorporated into the Si lattice. This corresponds to a value four orders of magnitude above the solid solubility limit of Se in Si. Further details on the advantages of using FLA for hyperdoping Si in lieu of PLM can be found elsewhere^22^. See also Methods for further information about FLA setup.

**Figure 1:**
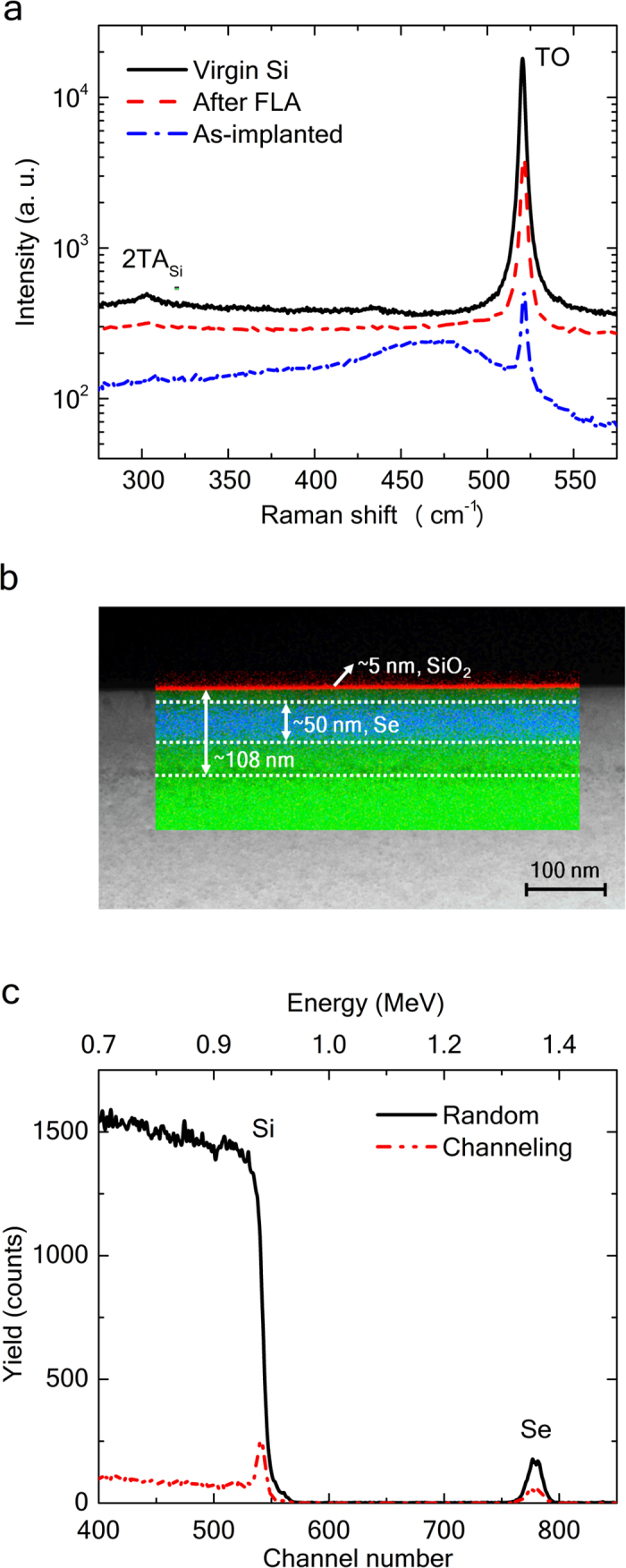
Microstructural properties. (**a**) μ-Raman spectra from virgin Si wafer, an as-implanted sample with 2.3% Se and the sample subsequently flash-lamp annealed for 1.3 ms. Y-axis has been offset for clarity. (**b**) Representative cross-sectional HAADF-STEM image combined with EDXS mapping for the same annealed sample (green: Si, red: O, blue: Se). (**c**) RBS/channeling spectra along the Si [100] crystallographic axis from the Se-implanted Si sample with 2.3% of peak Se concentration. The Si matrix is wholly recrystallized after 1.3 ms FLA and Se atoms have substituted the Si lattice sites.

Figure 1 summarizes the microstructural properties of the resulting Se hyperdoped Si layers. In particular, Fig. 1a depicts μ-Raman spectra from the as-implanted sample with 2.3% of peak Se concentration, after FLA and a virgin single-crystalline Si substrate used as reference. For the single-crystalline Si, the Raman peak at 303 cm^−1^ is ascribed to the second-order transverse acoustic phonon (2TA) scattering, whereas the one peaking at 520 cm^−1^ corresponds to the transverse optical (TO) phonon mode. A broad Raman band peaking at 460 cm^−1^ is observed in the as-implanted sample. This is connected with the amorphization process of Si during ion implantation^24^. The as-implanted sample also exhibits the 520 cm^−1^ sharp band, which arises from the Si substrate since, in amorphous Si, the penetration depth at 532 nm excitation laser is larger than that of the amorphous layer thickness (100 nm) formed during the implantation process. After FLA, only the sharp Raman band peaking at 520 cm^−1^ is exhibited, with no trace of the amorphous band. This fact proves the entire recrystallization of Se-implanted Si by FLA with relatively high quality.

Figure 1b shows a representative HAADF STEM micrograph obtained in Si [110] zone axis geometry combined with EDXS element maps for the indicated region (viz. green: Si, red: O, blue: Se). According to these results, there is a uniform Se distribution of around 50 nm in width within the recrystallized Si layer. Moreover, in contrast to gold-hyperdoped Si synthetized by PLM^3^, no surface Se segregation and no signs for nm-scale Se agglomerates despite the high Se concentration were observed. The sample surface is oxidized and the single-crystalline Se-hyperdoped Si contains some isolated stacking faults. End-of-range defects resulting from the implantation process are visible at a depth of around 108 nm.

Figure 1c exemplarily shows the typical RBS random and channeling spectra after FLA for the sample implanted with a fluence of 6 × 10^15^ cm^−2^. The RBS-channeling spectrum proves that the implanted layer is well recrystallized by FLA. Furthermore, it confirms the substitution of Se onto the Si lattice sites as previously reported^22^. A substitutional fraction of around 70% of Se atoms was roughly determined in all samples by the ratio between the channeling and the random spectra from the Se signal. Moreover, the RBS-channeling spectrum shows a near-surface minimum backscattered yield (ratio of the aligned to random yields) of about 4%, which is comparable to the one determined for the reference single-crystalline Si substrate.

On the other hand, room-temperature Hall effect measurements in van der Pauw geometry revealed effective electron concentrations of around (8.1 ± 0.7) × 10^19^ cm^−3^, (3.6 ± 0.7) × 10^20^ cm^−3^ and (2.8 ± 0.7) × 10^20^ cm^−3^ for the samples with Se concentrations of 3 × 10^20^ cm^−3^ (1.1%), 6 × 10^20^ cm^−3^ (2.3%), and 9 × 10^20^ cm^−3^ (3.5%), respectively. This provides a resulting effective electrical activation of Se dopants of around 27%, 60% and 30%, respectively. Interestingly, the effective electrical activation significantly decreases at the highest Se concentration. This is likely due to the formation of electrically inactive complexes such as Se-Se dimers, which give rise to the dopant deactivation^25^.

### SWIR absorption and band gap engineering

**Figure 2:**
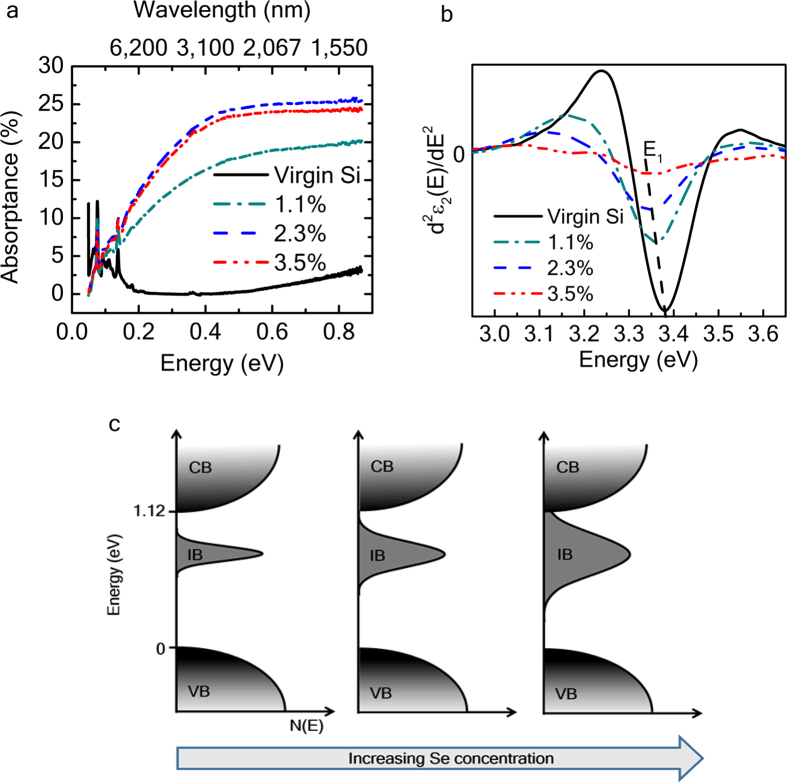
SWIR absorption and band gap engineering. (**a**) Optical sub-band gap absorptance spectra from FTIR measurements for virgin Si, and the three Se concentrations (1.1%, 2.3% and 3.5%). (**b**) Representation of the second derivative of the imaginary part of the dielectric function (*d*^*2*^*ε*_*2*_(*E*)/*dE*^2^) versus photon energy. E_1_ stands for the high-energy critical point of Si, which is red-shifted as Se concentration increases from 1.1% to 3.5%. (**c**) Sketch of the density of states and evolution of the impurity band as a function of increasing Se concentration. The IB merges with the conduction band above the metal-insulator transition.

We performed Fourier transform infrared spectroscopy measurements in order to inspect the SWIR absorptance from Se-hyperdoped Si layers for photon energies ranging from 0.04 to 0.86 eV (λ = 31,000 to 1,442 nm). Figure 2a depicts the absorptance (*A* = 1−*T*−*R*) computed by measuring transmittance (*T*) and reflectance (*R*) spectra for the three Se concentrations and a virgin Si substrate. Interestingly, all Se-implanted Si layers exhibit strong SWIR absorptance down to 0.4 eV (λ = 3,100 nm), which is not observable in the virgin Si substrate used as reference. Moreover, SWIR absorptance significantly increases with increasing Se concentration, except for the sample with highest Se content. This is attributed to the formation of Se-Se dimers since the Si host cannot any longer accommodate more Se atoms at such high concentrations (9 × 10^20^ cm^−3^). X-ray absorption fine structure (EXAFS) investigations have demonstrated that Se atoms relax from hyperdoped substitutional-type defects into precipitated SiSe_2_ states, which results in a decrease in the sub-band gap absorption due to the change in the chemical state of Se atoms^26^.

The modification of silicon’s electronic band structure as a function of Se content was investigated by spectroscopic ellipsometry at a grazing-incidence angle of 80° and over the photon energy range of 1–4 eV. Prior to the measurements, the native SiO_2_ layer was etched with aqueous HF to assure correct results. Figure 2b shows the second derivative spectra of the imaginary part of the dielectric function measured for the virgin Si substrate and hyperdoped Si samples with different Se concentrations. The structure of the dielectric function is connected with critical points of the electronic interband transitions. The ellipsometry results show a redshift of the critical point E_1_ in Si (interband transition along 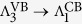 direction of the Brillouin zone)^27^ with increasing Se concentration (see Fig. 2b), which suggests direct modification of silicon’s electronic band structure involving a band gap narrowing.

This phenomenon is schematically represented in Fig. 2c by means of density of state diagram. The formation of an impurity band at high dopant concentrations has widely been studied^15^,^19^,^28^. In our case, for instance, as the Se atoms get closer to one another at high concentrations, their wavefunctions start to overlap, which results in broadening of the donor levels into an IB. The higher the Se concentration (*N*_*D*_), the broader the IB 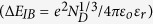
29, with Δ*E*_*IB*_ the IB width, *e* the elemental electron charge, *ε*_*o*_ the vacuum permittivity and *ε*_*r*_ the relative permittivity. When the IB is broad enough that it merges with the conduction band, electrons are no longer localized on the donor levels and become free carriers. At this point, the metal-insulator transition or Mott transition occurs^30^. This phenomenon typically takes place in Si at Se concentrations of around 3 × 10^20^ cm^−3^ 28. In addition to this broadening of the donor levels, the carrier distribution function also degenerates as the Se concentration increases. The binding energy of the Se atoms in Si has been reported to be in the range of 130–164 meV^31^.

### Room-temperature SWIR Si p-n photodiodes

**Figure 3:**
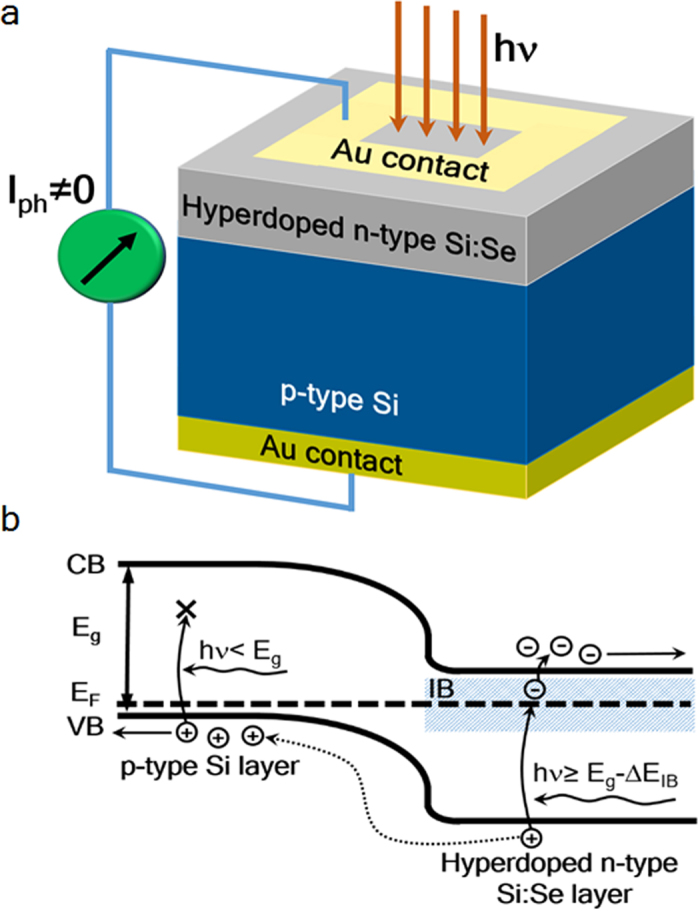
Device design and performance. (**a**) Cross-section scheme of the Se-hyperdoped Si p-n photodiode devices. (**b**) Band diagram of the impurity band-based Si p-n photodiode. Valence-band electrons with energies equal or higher than *hν* ≥ *E*_*g*_ − Δ*E*_*IB*_ are promoted to the IB from the valence band and in turn to the conduction band via thermally-mediated process. Created holes in the valence band are collected by the back Au contact, whereas conduction band electrons are evacuated by the top Au contact. As a result, the number of incident photons at allowed sub-band gap energies are proportionally converted into a measurable current. On the contrary, photons with energies lower than *hν* < *E*_*g*_ get unabsorbed at the p-type Si region.

We performed electrical contacts for interrogating the steady-state optoelectronic response of the devices. 5 mm^2^-photosensitive areas were defined by electron-beam evaporation of frame-like 1-mm-width Au contacts (Fig. 3a) atop the hyperdoped n-type Si:Se layers, whereas the 380-μm-thick p-type Si substrate was wholly covered by a 180 nm-Au thick layer providing the back contact. The cross-section of the p-n photodiodes is schematically presented in Fig. 3a.

The working principle of the room-temperature SWIR p-n photodiodes based on Se-hyperdoped Si is shown pictorially in Fig. 3b. Photons with energies *hν* ≥ *E*_*g*_ −∆ *E*_*IB*_ can solely be absorbed. Thus, these photons excite electrons from the valence band into the IB. Subsequently, electrons are thermally promoted into the conduction band since the IB extends close to the lower edge of the conduction band and energies comparable to the thermal energy (25 meV) are sufficient to trigger the thermally-mediated process. We experimentally verified the proximity of the IB to the conduction band by an Arrhenius plot from photocurrent measurements as a function of inverse temperature in the range of 100–300 K. We deduced activation energies of 38 ± 3 meV, 30 ± 3 meV and 35 ± 3 meV for Se contents of 1.1%, 2.3% and 3.5%, respectively. This indicates that the IB is not merged with the conduction band and thus the presence of a mini-band gap in between is inferred. The mini-band gap energy width is comparable to *k*_*B*_*T* = 25 meV.

**Figure 4:**
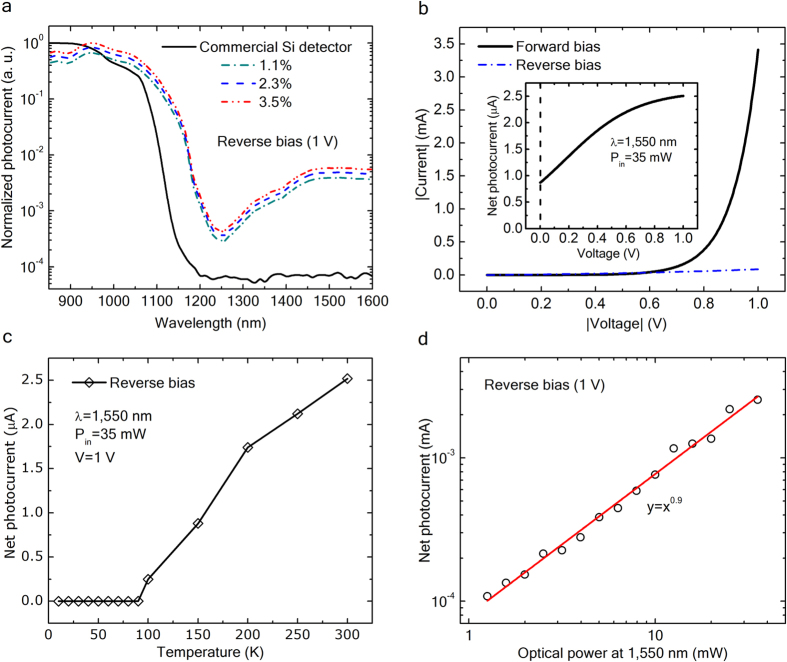
Steady-state photodetector characterization. (**a**) Normalized photocurrent of photodetectors for the studied atomic Se concentrations at reverse bias of 1 V. Spectral response of a commercial Si photodiode is shown for reference. All spectral photoresponses are normalized to the maximum Si photodiode response. (**b**) Typical room-temperature I-V curve of the photodiode at forward and reverse bias. Inset shows the net photocurrent versus voltage. (**c**) Net photocurrent as a function of temperature in response to the 1,550 nm illumination at reverse bias of 1 V. (**d**) Net photocurrent versus input optical power at a wavelength of 1,550 nm.

Figure 4a presents the spectral photoresponse of the devices at reverse bias of 1 V, using light from a 5 W Tungsten Halogen Lamp (Ocean Optics HL-2000 series) that is filtered by a monochromator. The area under illumination was always kept constant at approximately 4 mm^2^. We used a 780 nm long pass-filter in the wavelength range of 850–1,250 nm at the monochromator exit. There, we introduced an antireflection-coated Si wafer to eliminate additional contributions from second-order wavelengths longer than 1,100 nm. The spectral photoresponse of a commercial Si photodiode is shown for reference (see Fig. 4a, black solid line). The Se-hyperdoped Si photodetectors exhibit two well-defined broad photoresponse bands. The expected Si photoresponse above its band gap and a well-extended sub-band gap one – in close connection with FTIR results – are shown in Fig. 2a. The minimum photoresponse signal peaking at 1,250 nm stands for the mini-band gap between the upper edge of the impurity band and the conduction band edge which corroborates no IB merging with the conduction band in any of the three cases. The sub-band gap spectral signal shows an almost flat photoresponse above 1,450 nm. Moreover, the near-band gap spectral photoresponse of Se-hyperdoped Si photodetectors is extended about 80 nm deeper into the infrared region than that of the commercial Si photodiode. No photoresponse for energies below the 1.12 eV-Si band gap was observed for the commercial Si photodiode as expected. The spectral line-shape is also unaltered as Se concentration increases from 1.1% to 3.5%.

Hereafter, we present the results for 2.3% of peak Se concentration since no remarkable differences were observed from one sample to other and in turn to be consistent with the previously presented data. Figure 4b depicts a typical room-temperature current-voltage (I-V) characteristic of the photodiode at forward and reverse bias. The forward current exponentially increases with increasing forward voltage, whereas a reverse-current rectifying behavior takes place when the photodiodes are reversely biased. The dark current at 1 V reverse bias was measured to be 57.3 μA. In addition, the inset in Fig. 4b shows that the net photocurrent generated by 1,550 nm light linearly scales with increasing reverse voltage and subsequently saturates at 1 V. Net photocurrent stands for the subtraction between the photogenerated current under 1,550 nm illumination and dark current.

Temperature-dependent photoresponse measurements were carried out in order to explore more in depth the device’s optoelectronic performance. Experiments were performed over the temperature range of 10–300 K using a closed-cycle Helium gas cooling system. Devices were reverse biased at 1 V and optically illuminated at 1,550 nm keeping constant both the illuminated area and the input optical power. Figure 4c illustrates that the 1,550 nm net photocurrent significantly increases from 90 K to 300 K. This increase of the reverse-bias photocurrent as a function of the temperature corroborates a thermally-assisted process of electrons from the IB to the conduction band. This mechanism makes this class of photodetectors suitable for room-temperature operation in the SWIR spectral range. Alternatively, the reverse-bias net photoresponse remains at zero below 90 K. This is due to the electron freeze-out effect^32^, which means that at such low temperatures, the Fermi level is above the upper edge of the IB and no electrons from the IB are thermally promoted into the conduction band.

Figure 4d depicts the device photocurrent under reverse bias of 1 V as a function of the input optical power at 1,550 nm. We observe a linear sub-band gap photoresponse that suggests an IB-mediated single-photon absorption rather than a two-photon absorption mechanism. The responsivity, defined as R = I_ph_/P_in_, where I_ph_ is the generated photocurrent and P_in_ is the optical power entering the photodiode, was deduced to be 72 ± 3 μA/W at 1,550 nm wavelength excitation and 1 V reverse bias, whereas the external quantum efficiency (EQE) was determined to be approximately 6 × 10^−3^%. This EQE value is comparable to the one reported for Au-supersaturated Si photodiodes^3^. Moreover, the junction capacitance of the photodiodes was found to be around 53 nF/cm^2^ at 1 V reverse voltage considering an area of 1.2 × 10^−3^ cm^2^, whereas the rise time and the fall time were measured to be 7 ns and 23 ns, respectively. Since most of the photocurrent generation takes place in the Se-hyperdoped region of the device, the response time is expected to be mostly limited by the carrier diffusion to the depletion region, which makes this type of detectors suitable for applications in the MHz-speed range.

Based on the results from the first device’s demonstration, the device EQE is identified to be the crucial factor to be improved for practical applications. This might be addressed by including (i) antireflection coating for light trapping, (ii) efficient management of carrier extraction and/or (iii) developing thicker Se-hyperdoped Si layers. For the sake of illustration, we write the EQE as a function of the wavelength as follows:


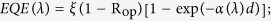


being *ξ* the fraction of electron-hole pairs that contribute to the photocurrent, R_op_ the optical power reflectance at the surface, *α(λ*) the optical absorption coefficient that has been reported to be 10^4^ cm^−1^ at 1,550 nm for Se-hyperdoped Si layers^12^, and *d* the active layer thickness. In particular, the factor 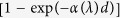 accounts for the fraction of the photon flux absorbed in the material. Based on the above relationship, it can be inferred that the total amount of absorbed light in the layer might be enhanced if the active layer thickness is increased from 50 nm to values larger than 1 μm (*d* > 1/*α, α* = 10^4^ cm^−1^, *d* > 1 μm). In the best-case scenario however, this alternative would only imply an improvement of a factor 5 on the EQE since the measured absorptance in the layers is of around 20%. Thus, the most promising and straightforward solution for the significant improvement of the EQE seems to be the suitable electrode design for the efficient carrier extraction since most of the generated electron-hole pairs recombine before being collected by the electrodes, strongly reducing the internal quantum efficiency, *ξ*(1 − R_op_). For this purpose, an interdigitated electrode arrays with an interdigital gap smaller than the minority carrier diffusion length should be conceived. Semitransparent polycrystalline silicon could also be used as electrode material instead of gold.

In summary, we demonstrated room-temperature SWIR p-n photodiodes from single-crystal Si hyperdoped with Se using a robust and reliable non-equilibrium processing consisting of ion implantation followed by millisecond-range FLA. This approach is repeatable, low-cost, allows for scalability and incorporates non-equilibrium Se concentrations as high as 9 × 10^20^ cm^−3^ in a way that the material’s pristine structure is preserved. It has also the advantage of preventing surface segregation, dopant redistribution and clustering. The SWIR photoresponse was demonstrated to be related with known Se deep-energy levels in Si. The photoresponse of hyperdoped Si can be extended up to 3,100 nm (0.4 eV) according to FTIR absorptance spectra. These photodetectors are also suited for simultaneously covering both visible and SWIR spectral range. We believe that this technology can start to be competitive once the conservative barrier of the 1% in quantum efficiency can be overcome.

## Methods

### Flash lamp annealing

The FLA system used in our experiments consists of an upper bank of twelve 30 cm long xenon (Xe) lamps spaced by about 3 cm, a wafer holder, a lower bank of halogen lamps allowing the wafer to be preheated to a desired temperature (a sort of rapid thermal annealing system), and two aluminum reflectors behind each bank of lamps. For effective annealing, FLA treated materials should have a high absorption coefficient in the spectral region of the Xe lamp. Silicon, for instance, is suited for the FLA processes. Wafers up to 200 mm in diameter can be processed with a lateral homogeneity better than 5%. Further details can be found elsewhere^22^,^23^.

### Material characterization

Microstructural properties of the resulting Se-hyperdoped Si layers were obtained by cross-sectional transmission electron microscopy (TEM) analyses which were performed with an FEI Talos F200X microscope operated at an accelerating voltage of 200 kV. In particular, atomic number contrast images were recorded in high-angle annular dark-field scanning transmission electron microscopy (HAADF-STEM) mode. For qualitative chemical analysis, energy-dispersive X-ray spectroscopy (EDXS) studies were done with a Super-X detector system attached to the Talos microscope. Prior to the TEM analysis, the specimen mounted in a dedicated double tilt analytical holder was placed for 15 s into a Model 1020 Plasma Cleaner (Fischione) to remove organic contamination. Classical cross-sectional TEM specimens were prepared by sawing, grinding, dimpling and final Ar ion milling.

Rutherford backscattering spectrometry/channeling (RBS/channeling) measurements were performed with a collimated 1.7 MeV He^+^ beam at a backscattering angle of 170° with the sample mounted on a three-axis goniometer with a precision of 0.01°. The channeling spectra were measured by aligning the sample to make the impinging He^+^ beam parallel with the Si [001] axis.

The phonon spectra were determined by μ-Raman spectroscopy in backscattering geometry in the wavenumber range of 100 to 600 cm^−1^ using a 532 nm Nd:YAG laser with a cryogenically cooled charge-coupled device camera attached to a spectrometer.

### Optical absorption characterization

The optical properties of the Se-hyperdoped Si layers were investigated by Fourier transform infrared spectroscopy in the SWIR spectral range and spectroscopic ellipsometry. Room-temperature SWIR transmittance and reflectance spectra were determined by using a Bruker Vertex 80ν FT-IR spectrometer equipped with a KBr beam-splitter and a mid-IR DLaTGS detector in the wavenumber range of 400 to 7,000 cm^−1^ (λ = 31,000 to 1,442 nm). Alternatively, for the ellipsometry measurements, a rotating analyzer ellipsometer VASE system (J. A. Woollam Co., Inc., USA) operating at photon energies ranging from 1 to 4 eV was used.

### Device characterization

Current-voltage characteristics were recorded with the help of a Keithley 237 source measurement unit, whereas capacitance-voltage measurements were performed by an Agilent HP 4284A precision LCR meter. Both rise and fall times were traced with a digital GHz Oscilloscope. A 1,550-nm laser was used as an excitation source, which was chopped with a 1,550-nm fiber-coupled acousto-optic modulator linked to a lock-in amplifier. A 50 Ω load resistance has been used in conjunction with a 50 Ω coaxial cable to ensure the best frequency response.

## Additional Information

**How to cite this article:** Berencén, Y. *et al*. Room-temperature short-wavelength infrared Si photodetector. *Sci. Rep.* **7**, 43688; doi: 10.1038/srep43688 (2017).

**Publisher's note:** Springer Nature remains neutral with regard to jurisdictional claims in published maps and institutional affiliations.
